# Virtual Learning Simulations in High School: Effects on Cognitive and Non-cognitive Outcomes and Implications on the Development of STEM Academic and Career Choice

**DOI:** 10.3389/fpsyg.2017.00805

**Published:** 2017-05-30

**Authors:** Malene Thisgaard, Guido Makransky

**Affiliations:** Department of Psychology, University of Southern DenmarkOdense, Denmark

**Keywords:** virtual learning simulations, e-learning, career development, education, STEM, social cognitive career theory

## Abstract

The present study compared the value of using a virtual learning simulation compared to traditional lessons on the topic of evolution, and investigated if the virtual learning simulation could serve as a catalyst for STEM academic and career development, based on social cognitive career theory. The investigation was conducted using a crossover repeated measures design based on a sample of 128 high school biology/biotech students. The results showed that the virtual learning simulation increased knowledge of evolution significantly, compared to the traditional lesson. No significant differences between the simulation and lesson were found in their ability to increase the non-cognitive measures. Both interventions increased self-efficacy significantly, and none of them had a significant effect on motivation. In addition, the results showed that the simulation increased interest in biology related tasks, but not outcome expectations. The findings suggest that virtual learning simulations are at least as efficient in enhancing learning and self-efficacy as traditional lessons, and high schools can thus use them as supplementary educational methods. In addition, the findings indicate that virtual learning simulations may be a useful tool in enhancing student’s interest in and goals toward STEM related careers.

## Introduction

Virtual learning simulations are increasingly being used in diverse educational and training contexts as a supplement to traditional educational methods (Bonde et al., 2014; Bric et al., 2015; Makransky et al., 2016a), and previous research has shown that they provide important educational benefits (Honey and Hilton, 2011). For instance, virtual learning simulations provide cost-effective access to state of the art training equipment and learning tools, beyond what many teaching institutions would be able to provide physically, due to financial or practical constraints (de Jong et al., 2013). Furthermore, simulations make it possible for students to grapple with realistic scenarios that may not be possible to experience in real life because they may be too dangerous or only occur rarely (Honey and Hilton, 2011; Smetana and Bell, 2012; Wang et al., 2014). A review of a selection of the literature to contrast the value of physical and virtual learning simulations, highlighted that virtual learning simulations let students observe otherwise unobservable phenomena, reduce time demand of experiments that would be very time consuming if done physically, and provide online, adaptive guidance (de Jong et al., 2013). By helping students develop an understanding of concepts and processes, through inquiry-based learning and engagement in realistic investigations, with continuous feedback, virtual learning simulations can foster learning in a new and innovative way (de Jong, 2006; Honey and Hilton, 2011; Furtak et al., 2012; Bonde et al., 2014). Specifically, learning simulations provide an excellent framework for engaging in inquiry-based learning, because they enable students to gain conceptual knowledge while independently investigating a scientific issue with the relevant methodology in the field. Furthermore, these simulations are able to motivate students by giving them challenges combined with continuous feedback in a learning environment that is more tailored to their individual learning needs and interests (Honey and Hilton, 2011).

Several previous studies have confirmed the educational benefits of virtual learning simulations. A critical review of the literature related to the use of computers in science instruction and learning, found that simulations can promote knowledge, develop relevant skills and facilitate conceptual change, just as effectively as traditional teaching methods (Smetana and Bell, 2012). This finding is further supported by a another systematic review and meta-analysis, where it was concluded that the use of simulations in science, does improve students’ science achievement compared to not using simulations (D’Angelo et al., 2013). Furthermore, Bonde et al. (2014) found that virtual learning simulations increased subject knowledge significantly as a stand-alone tool, and even more so in combination with a traditional teaching method (in this case a lecture), when tested on college students. Also, the study revealed that the students themselves found the simulations helpful in increasing their self-reported motivation, interest and learning (Bonde et al., 2014). Adding to this, Makransky et al. (2016a), found that virtual learning simulations increased student’s intrinsic motivation, in addition to learning and self-efficacy.

Although virtual learning simulations have shown great promise, there are still many unanswered questions regarding their potential. For instance, few studies have investigated the non-cognitive outcomes related to using virtual learning simulations in high school populations. Furthermore, little is known about how virtual learning simulations can influence students’ interest in science, technology, engineering and mathematics (STEM) fields. Therefore, the present study sets out to fill this gap by comparing simulations and traditional lessons on non-cognitive skills in the form of motivation and self-efficacy in addition to cognitive outcomes. This is done out of appreciation for the growing evidence establishing their importance in predicting academic success and subsequent positive occupational outcomes (Heckman et al., 2006; Heckman and Kautz, 2012; Garcia, 2014). Furthermore, we investigate if virtual learning simulations can serve as a catalyst for STEM academic and career development, through their impact on self-efficacy and outcome expectations, based on social cognitive career theory.

Importantly, the present study does not set out to provide evidence for the superiority of the virtual learning simulations, but rather provide evidence of the comparability of effectiveness of the simulations to traditional lessons. Many studies have confirmed that the simulations should be used as a supplement to traditional teaching methods, not as a replacement for them (Smetana and Bell, 2012; de Jong et al., 2013; Wang et al., 2014; Makransky et al., 2016b). de Jong et al. (2013) highlighted that the combined use of physical and virtual learning offer benefits that none of them can as a standalone method. Thus, the present study aspires to provide evidence, to serve as the empirical foundation for using the simulations as a supplementary teaching method in high school education.

The intention to provide evidence for simulations efficacy in high school, is summarized in the first research aim of the present study: *to examine if virtual learning simulations are effective in bringing about learning benefits for high-school students, both cognitive and non-cognitive, in comparison to traditional lessons.*

Learning and improvement of non-cognitive skills are potential important immediate effects of the virtual learning simulations, but there may also be possible long term benefits of using virtual learning simulations. The simulations may be a useful tool in changing young people’s interest and goals related to STEM studies and careers. There is currently a challenge in making STEM studies and careers attractive to young people (The Business-Higher Education Forum, 2011). This is a problem because these fields are growing areas of work and research and necessary for building a strong economy in the future (Honey and Hilton, 2011; The Business-Higher Education Forum, 2011). Therefore, it is important to attract many more highly skilled workers with advanced knowledge and expertise to these fields, to keep up with the development in this area (European Commission, 2004). In order to meet this need, it is necessary to find new ways to generate interest among young students in these areas of work. Virtual learning simulations constitute one possible way of doing this.

The social-cognitive career theory by Lent et al. (1994), is used as a comprehensive theoretical framework to understand how learning simulations may influence students’ academic and career interests and choices in this study. The theory proposes a variety of constructs and mechanisms relevant to academic and career development.

**FIGURE 1:**
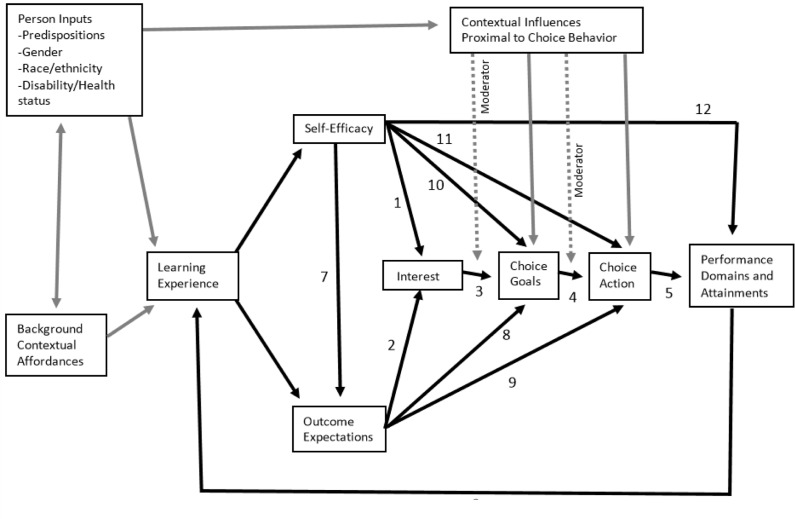
**Visual representation of the components of the model in social cognitive career theory and their interactions with each other (Lent et al., 1994, p. 93)**.

According to the theory, learning experiences influence academic and career development largely through the cognitive mechanisms self-efficacy and outcome expectations (see **Figure 1**; Lent et al., 1994). Through a learning experience a person enhances their capabilities, establishes their own standard of performance, forms a judgment of their own self-efficacy, as well as expectations about what outcomes this performance may lead to. As such, a learning experience may not directly influence academic and career development, but instead influences the person’s self-efficacy appraisals and outcome expectations, which in turn influence interest and subsequent goal choices and actions. Interest in a certain activity is developed when a person has high self-efficacy and positive outcome expectations (see paths 1 and 2 in **Figure 1**; Lent et al., 1994). In other words, people will have an enduring interest in activities where they deem themselves competent and hold positive expectations about the outcomes of these activities. Interest in turn promotes choice goals, and subsequent choice actions (see paths 3 and 4 in **Figure 1**; Lent et al., 1994). This means that a person will form concrete intentions and aspire to pursue a certain academic or career path, when they have a primary interest in it, and that these intentions increase the likelihood of concrete actions in the pursuit of this academic path or career.

A variety of empirical evidence has confirmed the links between concepts and predictions made in the theory. A study testing the social cognitive career theory with middle school students in math and science yielded results in support of the theory’s prediction that vocational interests are reflective of students’ concurrent self-efficacy and outcome expectations, as well as their link to choice goals and action (Fouad and Smith, 1996). A similar study, examining the application of the theory with a sample of students at university, also found results supporting the relationships between the mechanisms in the theory (Diegelman and Subich, 2001). Specifically, they found that self-efficacy and outcome expectations for a specific degree related significantly and positively to interest in, and intent to pursue that degree and that both mechanisms accounted for significant incremental variance in predicting interest and intent to pursue the degree.

In relation to the development of STEM interest, the social cognitive career theory predicts that students will develop an interest in STEM subjects and careers, if they have high self-efficacy and positive outcome expectations about them. Virtual learning simulations could potentially serve as the learning experience, from which students’ judge their self-efficacy and form their outcome expectations. In line with the theoretical framework, the simulations should be effective in increasing interest, because they have been found to increase self-efficacy through positive learning experiences and performance feedback. Whether or not the simulations have a positive influence on outcome expectations is unclear because we found no previous research that has investigated this. However, because the virtual learning simulations depict a realistic laboratory and utilize cases mimicking real world work situations, they may help to give the students a positive experience with the area of work, thereby increasing their positive expectations toward it. Supporting this idea, Bandura (1999) stated in an article on his social cognitive theory that “Electronic technologies greatly extend human capabilities to test the likely outcomes of given decisions and courses of action through the use of computerized enactments in simulated realities without having to carry out the activities” (Bandura, 1999, p. 27).

In addition to the possible effects on self-efficacy and outcome expectations in general, the more specific nature of the outcome expectations that a learning experience may influence, is of important consideration as well. Bandura (1986) described several aspects and classes of outcome expectations. Lent et al. (1994) emphasize three of these classes as possibly important to career behavior. These are physical, social, and self-evaluative. Physical outcomes are material in nature, such as monetary rewards. Social outcomes relate to the social consequences of an action, for example approval/disapproval from significant others or helping others. Self-evaluative outcomes relate to individuals appraisals of themselves, for example self-satisfaction, mastery and fulfillment of personal standards. It is possible that they influence interest differently and to varying degrees, which means that the learning experience in question (in this case a simulation), should aim at influencing the type of outcome expectations most important to interest.

The idea that simulations could promote interest in pursuing STEM careers, has already found some preliminary support from a survey on the subjective appraisals of learning simulations, where high school students indicated that they increased their interest in pursuing STEM careers (Bonde et al., 2014). This is concordant with the idea that adolescents and young adults, have the most fluid interest development (Lent et al., 1994), highlighting that high-school students may be the ideal target for interventions aiming to enhance interest in a specific field. However, more solid empirical evidence and theoretical considerations are needed to determine if simulations are a viable method to increase students’ interest in and pursuit of STEM studies and careers.

Virtual learning simulations may constitute a new potential tool for influencing young people’s academic and career development, toward STEM areas, thereby meeting the demands of future society. In the present study, the viability of using learning simulations as such a tool is investigated. This is done by investigating if the virtual learning simulation influences students’ academic and career development, based on its ability to influence self-efficacy and outcome expectations according to social cognitive career theory. By looking closer at the factors and mechanisms related to academic and career development proposed by the social cognitive career theory, and how virtual learning simulations fit into this, we may enhance our understanding of the usability and efficacy of the simulations. This leads to the second aim of the present study: *to examine whether virtual learning simulations can serve as a catalyst for STEM academic and career development, through its impact on self-efficacy and outcome expectations.*

In summary, the present study explores the use of virtual learning simulations in a high school setting with two specific research aims, targeted at the benefits of its use here and now, and as a tool in the development of future academic and career choice. In extension of the two research aims, three specific research questions were posed:

*Research question 1:* Do students who use a virtual learning simulation have comparable learning outcomes to students receiving a traditional lesson in the same subject?

*Research question 2:* Do students who use a virtual learning simulation have comparable non-cognitive (self-efficacy and intrinsic motivation) and career related outcomes (interest and outcome expectations) to students receiving a traditional lesson in the same subject?

*Research question 3a:* Do self-efficacy and outcome expectations predict students’ interest in STEM related tasks?

*Research question 3b:* Which of the three subdomains of outcome expectations (physical, social, and self-evaluative) predict on interest in biology?

## Materials and Methods

### Participants

The sample consisted of 128 students from 4 different high schools in Denmark. There were 88 females and 40 males, with an average age of 18.2 years. The participants were attending either their second (72) or third (56) years of high school, taking either biology or biotechnology as an elective medium or advanced level subject (41 B-level (medium) and 87 A level (advanced).

The study was conducted according to the Helsinki Declaration (Williams, 2008) and participation in the experiment was voluntary. All participants were given written and oral information describing the research aims and the experiment before participation. Written consent was gathered from all participants and their parents if they were younger than 18 years old, in accordance with the ethical regulations of the health research ethics committee in Denmark. Finally, the study protocol was submitted to the Regional Committee on Health Research Ethics for Southern Denmark, who indicated that no written consent was required by Danish law.

### Procedure

The study used a crossover repeated measures design, were all participants used the virtual laboratory simulation on a desktop computer, as well as receiving a traditional lesson in the same subject as a control. The participants were grouped as whole classes, and each class was assigned as either group A or group B. This was done to ensure high similarity to their everyday classes. Ecological validity was thus prioritized to random assignment. During step 1 of the trial, group A (66 participants) used the virtual learning simulation and group B (62 participants) received a traditional lesson in the same subject, prepared by their teacher. This step took approximately 1.5 h. After a short lunchbreak, step 2 of the trial began, and the two groups switched conditions, so that group B used the virtual learning simulation and group A received a traditional lesson, again having a duration of approximately 1.5 h. All sessions took place within regular school hours. The students answered a questionnaire containing a battery of tests and scales at three different times during the trial; before step one (pre-test), immediately after step 1 (midway-test), and immediately after step 2 (post-test).

### Materials

The virtual learning simulations used in this study was developed by Labster (2016a). Labster has developed a range of virtual laboratory simulations within the subjects of biology and biotechnology. The virtual laboratories contain several pieces of advanced lab equipment, such as a PCR-machine and a gel-electrophoresis machine, as well as learning tools and 3D visualizations that are only available in a simulated environment. For example a visualization of the duplication of DNA occurring within a PCR-machine or a visual rendering of how a gel-electrophoresis machine turns a DNA sample into a unique DNA profile. Through the work with a realistic case containing a central problem, users use the equipment in the virtual lab to explore a specific area of biology or biotechnology, learning about the relevant concepts and methods, thereby finding a solution to the case problem. To support the scientific exploration, the simulations feature in depth theoretical wikis, with descriptions of relevant theory and background information. Throughout each simulation, users respond to multiple-choice questions to check if they have learnt the material and are ready to continue on to the next task. Students can only progress in the simulation, when they are able to answer correctly, ensuring that they utilize the theory and experiments fully instead of simply racing through the simulation.

The simulation that is used in the present study was designed to fit the educational level of a high-school curriculum. The simulation concerns the topic of evolution, requiring the user to work with virtual lab equipment to identify an unknown animal found on a beach, thereby exploring various aspects of the subject and scientific concepts, such as natural selection and genetics. It features videos, depictions of genetic relationships, and a 3D visualization of the growth of a population of a species on an island (see Labster, 2016b for a video presentation of the evolution lab, and Appendix A for a screenshot from the lab). The case in the simulation is designed to represent a realistic situation, where users are presented with different challenges and multiple-choice quizzes throughout the simulation. The majority of the students were able to complete the simulation in between 30 min and 1.5 h. The students used their own computers to play the simulation.

As a control the students received a traditional lesson in evolution, prepared by their own teacher. The lessons took approximately 1.5 h. The lessons were prepared by the student’s own teacher to attain a control as similar as possible to the lessons they usually receive. This was done to make sure that we could make a direct comparison between the virtual learning simulations and the educational methods the students are used to, thereby being able to give a more realistic picture of how the simulations can work as a supplement to regular lessons. Preceding the teachers’ preparation for conducting the lesson, each teacher was given access to the virtual learning simulation and given the questions that were used to assess the students learning outcomes. They were instructed to base their lesson on the information given in the simulation and prepare the students as best as possible for the learning outcome questions, using the educational methods they usually use in a traditional lesson. Because each high school had a different teacher connected to the study, the specific lesson they received varied somewhat in the educational method(s) chosen. The different methods used were group work, reading a text, presenting a topic to the class and exploring the topic based on a worksheet, experiments, as well as some extent of teacher presentation of the subject (see Appendix B for a description of the approach taken by each high school included in the study).

All questionnaires were set up and administered using SurveyMonkey (2016). The IBM SPSS software package was used to conduct data analysis and investigate the research questions in the study (IBM, 2016). The psychometric properties of the items and scales in the study were assessed in the RUMM 2030 software package (Andrich et al., 2010).

### Measures

In line with previous research regarding virtual learning simulations, as well as the social-cognitive career theory, the constructs that the students were assessed on were learning (knowledge about evolution), self-efficacy, intrinsic motivation, outcome expectations (about a job within biology/biotechnology) and interest (interest in biology related tasks). All questions and scales were administered in Danish to accommodate the high-school students’ language abilities. All scales that were originally in English, were adapted from English to Danish by translating and back translating in accord with the international test commission guidelines for translating and adapting tests (International Test Comission, 2010).

The learning outcome was assessed by measuring the students’ knowledge of evolution. Based on the content of the virtual learning simulation, 20 multiple choice questions were developed to assess their knowledge. Prior to testing all knowledge questions were reviewed and approved by a high-school biology teacher to ensure that the difficulty was appropriate.

Self-efficacy was assessed using eight items adapted from the Motivated Strategies for Learning Questionnaire on a scale from 1 to 7 (Pintrich et al., 1991). Intrinsic motivation was assessed using five items adapted from the Interest/Enjoyment Scale from the Intrinsic Motivation Inventory on a scale from 1 to 5 (Deci et al., 1994).

To measure students’ interest in biology related tasks they were asked to indicate their degree of interest (from 1, very low to 5, very high) in performing six activities common to work within the field of biology. This way of measuring interest was chosen in light of previous research using this method to assess interest in computing disciplines (Lent et al., 2008, 2011). The six activities used in the scale were based on tasks performed by professionals working within biology related occupations according to the O^∗^Net online database of occupations (Honey and Hilton, 2011). The specific occupations used were biologist, geneticist, as well as zoologists and wildlife biologists. These were chosen because they had the best fit with the specific topic examined in this study; evolution.

To measure students’ outcome expectations regarding a career within the field of biology, a scale was developed based on Banduras definition of outcome expectations (Bandura, 1986; Lent et al., 1994). A total of 15 items were developed to assess the physical, social and self-evaluative aspects of the concept; 5 items for each sub dimension on a scale from 1 to 5. The items were developed based on their importance to each sub-domain and subsequently reviewed by a subject matter expert within organizational psychology to ensure that each item conveyed realistic aspects of work life. All items were positively worded so that a high total score would indicate high positive outcome expectations.

## Results

The psychometric properties of all the scales were evaluated before investigating the research questions. Fit to the Partial Credit Model (PCM; Masters, 1982) within the framework of Item Response Theory (Embretson and Reise, 2000) was assessed for each scale (for more information about validating scales using the PCM see: Pallant and Tennant, 2007; Makransky and Bilenberg, 2014; Makransky et al., 2014, 2017). The results showed that the self-efficacy, intrinsic motivation, interest, and outcome expectations scales all fit the PCM. All scales also had satisfactory reliability (average Cronbach’s alpha reliability across three time points: Self-efficacy: 0.94; Intrinsic Motivation: 0.76; Interest: 0.71; Outcome expectations: 0.93). One-way between subjects ANOVAs were conducted to investigate if there were differences between the high schools on the effect of the lessons for all of the outcome variables in the study. That is, we compared the students’ change scores from prior to the lesson to right after the lesson (irrespective of whether the lesson was the first or the second intervention) for the variables of knowledge of evolution, self-efficacy, motivation, interest, and outcome expectations. No significant differences were found across the four high schools for gains in knowledge of evolution, self-efficacy, motivation, interest or outcome expectations, indicating that the results from the high schools could be pooled in subsequent analyses.

### Research Question 1

**FIGURE 2:**
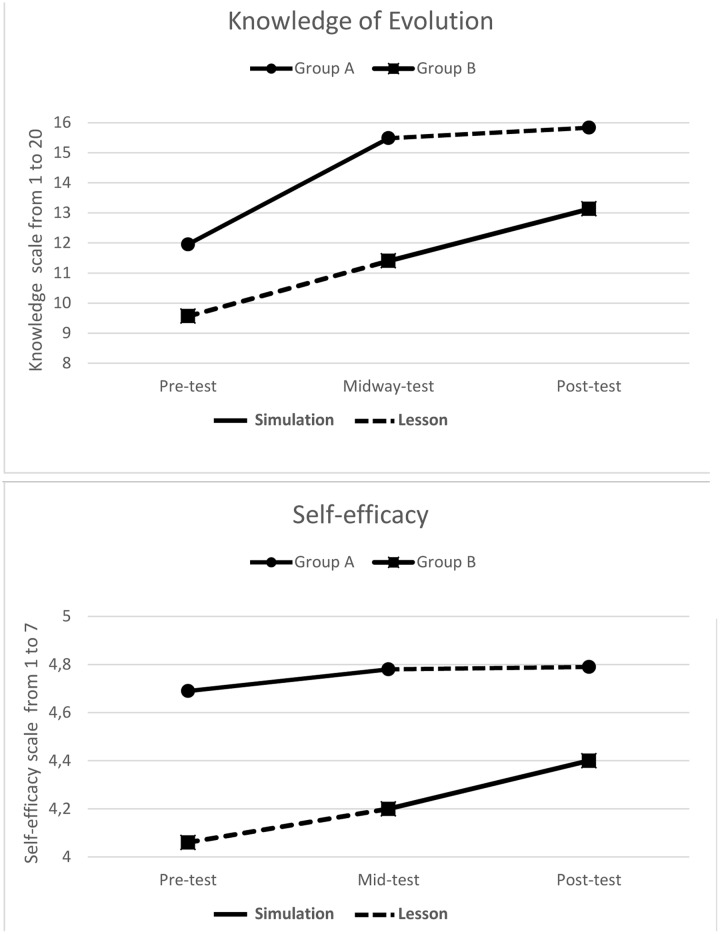
**Change in knowledge of evolution in the top panel, and self-efficacy in the bottom panel across the pre/mid/and post-test for the two groups of students**.

Research question 1 was investigated with a two group (group A/B) by three time points (pre/mid/post-test) mixed model ANOVA. The results showed a significant group by time interaction for knowledge of evolution [*F*(2) = 9.69, *p* < 0.001]. Based on the significant results, *post hoc* tests were used to investigate the results further (see top panel **Figure 2** for a visual overview of the results). There was a significant learning outcome (increase in knowledge of evolution) from the pre- to the mid-test following the simulation [*M* = 11.95 to *M* = 15.48; *t*(65) = -11.80, *p* < 0.001, *d* = 1.45] and lesson [*M* = 9.56 to *M* = 11.40; *t*(65) = 5.83, *p* < 0.001, *d* = 0.65]. Furthermore, group A who used the simulation had a significantly higher change (*M* = 3.53) than group group B who used the lesson [(*M* = 1.84), *t*(126) = -3.89, *p* < 0.001; *d* = 0.69] between the pre- and mid-test.

When adding the lesson to the simulation group (group A), no significant further increase in knowledge was found [*M* = 15.48 to *M* = 15.83; *t*(65) = -1.75, *p* = 0.086, *d* = 0.14]. When adding the simulation to the lesson group (group B), a further significant increase in knowledge was found [*M* = 11.40 to *M* = 13.13; *t*(61) = -5.59, *p* < 0.001, *d* = 0.59]. Furhtermore, group B who used the simulation at step 2 had a significantly higher change (*M* = 1.73), than group A who used the lesson at step 2 [*M* = 0.35), *t*(126) = 3.80, *p* < 0.001; *d* = 0.67].

**FIGURE 3:**
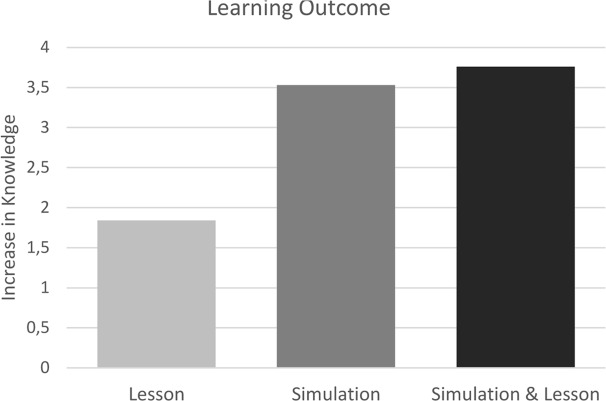
**Increase in knowledge after a lesson, simulation and both methods combined.** Increase in knowledge is the mean difference in correct answers in a 20-item multiple-choice quiz from before to after the intervention, or before to after both interventions.

An independent samples *t*-test comparing the change scores from pre-test to post-test for the two groups was conducted, to examine if there was an order effect of the two interventions. There was no significant difference in change scores for group A (*M* = 3,88) and group B [*M* = 3.56), *t*(126) = -0.740, *p* = 0.461; *d* = 0.13]. Because no order effect could be found, increase in knowledge from pre-test to post-test of the two groups were calculated collectively. A paired samples *t*-test of pre-test and post-test scores, showed a significant increase in knowledge from a mean of 10.80 on a scale from 0 to 20 in the pre-test to 14.53 in the post-test *t*(127) = -17.60, *p* < 0.001; *d* = 1.28. Finally, a paired samples *t*-test showed that the change in knowledge from right before to right after the simulation (*M* = 2.66) was significantly higher than the change in knowledge from right before to right after the lesson (*M* = 1.07), *t*(127) = -4.31, *p* < 0.001; *d* = 1.32 (see **Figure 3**).

### Research Question 2

Research question 2 was also investigated with a two group (group A/B) by three time points (pre/mid/post-test) mixed model ANOVA for the non-cognitive variables self-efficacy, intrinsic motivation, interest and outcome expectations. The results showed a significant group by time interaction for self-efficacy [*F*(2) = 5.55, *p* = 0.004], but no significant group by time interactions for motivation [*F*(2) = 1.64, *p* = 0.849], interest [*F*(2) = 1.00, *p* = 0.368], or outcome expectations [*F*(2) = 0.07, *p* = 0.932].

Based on the significant results for self-efficacy, *post hoc* tests were used to investigate the results further (see bottom panel **Figure 2** for a visual overview of the results). There was not a significant increase in self-efficacy from the pre- to the mid-test following the simulation [*M* = 4.69 to *M* = 4.78; *t*(65) = -1.80, *p* = 0.076, *d* = 0.12] but the increase was significant following the lesson [*M* = 4.06 to *M* = 4.20; *t*(61) = -2.74, *p* = 0.008, *d* = 0.10]. However, the difference between the change scores for these two groups were not significant *t*(126) = 0.69, *p* = 0.494; *d* = 0.12, between the pre- and mid-test.

When adding the lesson to the simulation group (group A), no significant further increase in self-efficacy was found [*M* = 4.78 to *M* = 4.79; *t*(65) = -0.22, *p* = 0.83, *d* = 0.01]. When adding the simulation to the lesson group (group B), a further significant increase in self-efficacy was found [*M* = 4.2 to *M* = 4.4; *t*(61) = -4.04, *p* < 0.001, *d* = 0.17]. Furthermore, group B who had the simulation at step 2 had a significantly higher change (*M* = 0.20), than group A who had the lesson at step 2 [(*M* = 0.01), *t*(126) = 3.19, *p* = 0.002; *d* = 0.56].

**FIGURE 4:**
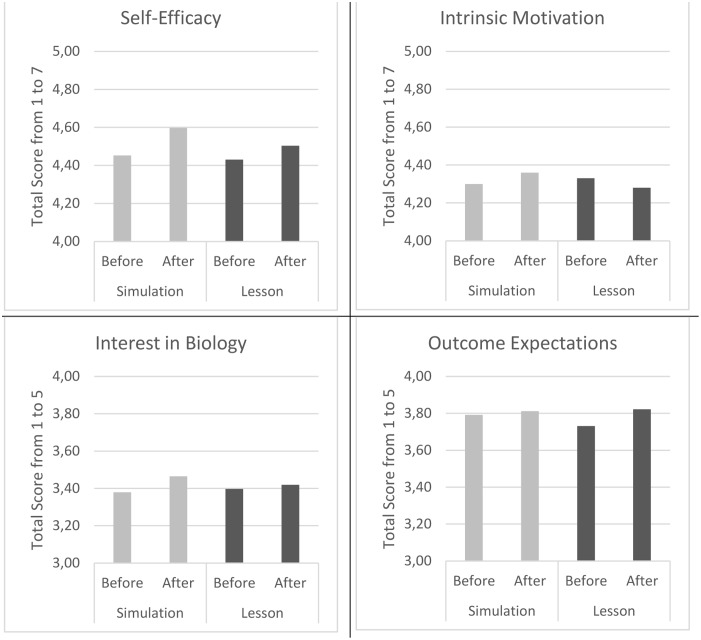
Change in self-efficacy, intrinsic motivation, interest and outcome expectations regarding a career within the field of biology, following the simulation and lesson interventions.

A final analysis for self-efficacy was conducted to compare the overall results of the simulation and the lesson irrespective of the order in which the intervention was administered (see top left panel of **Figure 4**). Results of the paired samples *t*-tests on the combined scores, showed a significant increase in self-efficacy from a mean of 4.45 on a scale from 1 to 7 before the simulation to 4.60 after the simulation [*t*(127) = -4.023, *p* < 0.001; *d* = 0.14]. Similarly, results showed a significant increase in self-efficacy from a mean of 4.43 before the lesson to 4.50 after the lesson [*t*(127) = -2.318, *p* = 0.022; *d* = 0.06], however, this result was not sigificatn after accounting for multiple tests with a Bonferroni correction.

Since the results for the mixed model anova were not significant for the variables intrinsic motivation, interest, and outcome expectations only the overall results of the simulation and lesson irrespective of the order of the intervention were conducted (see **Figure 4**). Results showed no significant increase in instrinsic motivation for biology from a mean of 4.30 to 4.36 [*t*(127) = -1.149, *p* = 0.253; *d* = 0.08] from right before to right after the simulation, and a non-significant decrease from right before to right after the lesson from a mean of 4.33 to 4.28 [*t*(127) = 1,258, *p* = 0.211; *d* = 0.07].

A significant increase in interest in biology related tasks was found from a mean of 3.38 to 3.46 [*t*(127) = -2.301, *p* = 0.023; *d* = 0.14] from rigth before to right after the simulation. However, results showed no significant change in interest in biology related tasks from right before to right after the lesson from a mean of 3.40 to 3.42 [*t*(127) = -0.776, *p* = 0.439; *d* = 0.03). Finally, there was no significant increase in outcome expectations related to a biology career from right before to right after the simulation from a mean of 3.79 to 3.81; [*t*(127) = -0.722, *p* = 0.471; *d* = 0.03], or the lesson from a mean of 3.72 to 3.82; [*t*(127) = -1.271, *p* = 0.206; *d* = 0.17].

A paired samles *t*-tests comparing the effect of the two different conditions showed no significant differences between the two conditions for gains in self-efficacy [*t*(127) = -1.484, *p* = 0.140], instrinsic motivation [*t*(127) = -1.734, *p* = 0.085], interest [*t*(127) = -1.127, *p* = 0.262], or outcome expectations [*t*(127) = 1.234, *p* = 0.220]. This means that the increase in non-cognitive variables that students gained from the simulation and the lesson did not differ significantly.

### Research Questions 3a and 3b

To investigate research question 3a, a stepwise multiple regression was used to assess the ability of two biology career related measures (self-efficacy in biology and outcome expectations toward a biology related career) to predict levels of interest in biology related tasks, as well as self-efficacy’s contribution to outcome expectations in accord with the social cognitive career theory. Because the theory explains how self-efficacy and outcome expectations contribute to interest following a learning experience, the analysis was performed on post-test results.

First, a standard linear regression was performed to determine self-efficacy’s contribution to outcome expectations. A significant regression equation was found when predicting outcome expectations based on self-efficacy *F*(1,126) = 16.436, *p* < 0.001, with an *R*^2^ of 0.12.

The second analysis was a stepwise multiple regression with outcome expectations and self-efficacy as predictors of interest in biology (that is Edges 1 and 2 in **Figure 1**). Outcome expectations was included in the first step of the analysis because it had a higher beta than self-efficacy in predicting interest in biology. At step 1 outcome expectations was significantly related to interest [*F*(1,126) = 20.549, *p* < 0.001; *R*^2^ = 0.14]. There was a significant increase in *R*^2^ after adding self-efficacy to the model [*F*(2,125) = 13.526 *p* < 0.001; *R*^2^ = 0.18]. This means that including both self-efficacy and outcome expectations, explains 18% of the variance in interest in biology related tasks.

Finally, a stepwise multiple regression was performed to determine the contribution of the three subdomains of outcome expectations (physical, social, and self-evaluative) on interest (research question 3b). At step 1 of the analysis the self-evaluative subdomain entered into the regression equation and was significantly related to interest [*F*(1,126) = 15.265, *p* < 0.001; *R*^2^ = 0.11]. The physical and social subdomains did not add a significant amount of incremental validity to the equation at step 2 of the analysis. This means that the self-evaluative subdomain was the only subdomain of the three that had a significant unique contribution to interest.

## Discussion

The results of this study indicate that virtual learning simulations are superior in promoting learning, and are comparable in promoting non-cognitive (self-efficacy and intrinsic motivation) and career related outcomes (interest and outcome expectations) when compared to a traditional lesson. In addition, the findings indicate that learning simulations hold a potential to influence students’ academic and career development, through their effect on self-efficacy.

### Cognitive and Non-cognitive Outcomes of the Two Interventions

The results of this study showed that both the simulation and the traditional lesson led to significant increases in subject matter learning, in the form of knowledge of evolution. This increase was significantly larger following the simulation intervention, compared to the lesson intervention. The combination of both simulation and lesson brought a further increase in knowledge; however, this was only significant when the lesson was first, followed by the simulation. Still, no order effect was found when comparing the two groups’ results from pre- to post-test. These findings are important because they suggest that simulations can be at least as capable, if not more so, in enhancing learning, compared to traditional lessons. This means that teachers can use the simulations as a supplement to their lessons, knowing that they will not be compromising on the students learning outcomes. They can use the two as a powerful combination of learning tools, in line with previous findings, recommending the use of simulations as a supplement to traditional teaching methods (de Jong et al., 2013; Wang et al., 2014; Makransky et al., 2016b). Furthermore, the findings provide support for the current move toward more technology based learning methods (Peters and Araya, 2011), and are an empirical extension of previous evidence supporting the learning gains from virtual learning simulations (e.g., Bonde et al., 2014; Makransky et al., 2016a,b).

In addition to the confirmatory results regarding learning, the present study was also able to show interesting changes in the non-cognitive variables tested. The simulation led to a small significant increase in self-efficacy. However, neither the simulation nor the lesson had any significant influence on intrinsic motivation or outcome expectations. Finally, the simulation significantly increased interest in biology related tasks, where the lesson did not. Importantly, analysis revealed that the increases in all non-cognitive variables gained from either intervention, did not differ from each other significantly, indicating that neither was superior to the other. As with gains in knowledge, these results confirm that the learning simulation is at least as effective as the traditional lesson in enhancing students’ non-cognitive skills.

The finding that the simulation was able to increase self-efficacy, is in line with previous research on learning simulations (Bonde et al., 2014; Makransky et al., 2016a), and adds to the value of using the simulations as a teaching method in high-schools. However, the present study was not able to show all the same benefits of learning simulations as previous research has. In their most recent study examining learning simulations Makransky et al. (2016a) were able to find a significant increase in intrinsic motivation, following a learning simulation in a university sample. The present study was not able to confirm this finding in a high school sample. Theoretically, the simulations should be able to improve student motivation by giving them a sense of autonomy and increasing their overall competency in the subject (Ryan and Deci, 2000) and the findings from Makransky et al. (2016a) confirms that this link is at least plausible. Therefore, the lack of effect on intrinsic motivation from the simulation in the current study may not necessarily be reflective of the simulations inherent ability to do so, but instead be due to methodological considerations. It is possible that inducing an increase in motivation is easier in a university sample, simply because the lectures they are used to, where passive listening is the norm, may be very different from the simulations and the simulations therefore are more motivating in comparison. In contrast, high school students usually receive much more varied and involving lesson types and the simulation therefore may not be equally conducive of motivation for them, because they are not much different from what they are used to. However, the most likely cause of the difference may be a sampling error, where the sample tested in the present study is slightly different than the one tested in the study by Makransky et al. (2016a). The two studies achieved somewhat different effect sizes (*d* = 0.24 compared to *d* = 0.08 in the present study). Still, the difference is a rather small one overall, corroborating the idea that a sampling error is the cause. In light of this, more research is needed to determine the relationship.

### Academic and Career Development with Learning Simulations

Results of this study were able to confirm the links between cognitive mechanisms related to academic and career choice, proposed by the social cognitive career theory. Self-efficacy in biology was significantly able to predict positive outcome-expectations regarding a biology related career, and both self-efficacy and outcome expectations significantly predicted interest in biology related tasks. These findings enable us to make certain assumptions about the possible effects of the learning simulations role in academic and career choice. Because we were able to confirm sections of the theory based on our results, we can also with caution, suggest that the additional links and predictions made by the theory will be applicable to our study.

The simulation did significantly increase self-efficacy, which should lead to an increase in interest, which the study also found. However, this very small immediate increase in interest may not be the end of the simulations effect. The social cognitive career theory states that there may be a temporal lag between newly acquired self-efficacy and interest (Lent et al., 1994), this means that the full effect on interest may not be present immediately, but instead will increase over time, when self-efficacy has been strengthened on multiple occasions. Furthermore, the relatively small increase in self-efficacy following one use of the simulation may not have been sufficient to promote interest in students with a low level of interest to begin with, due to a threshold effect, requiring at least moderate levels of self-efficacy to promote the formation of interest (Lent et al., 1994). Finally, there is a theoretical possibility that the two cognitive mechanisms, self-efficacy and outcome expectations, interact in such a way that self-efficacy may only result in interest, when a person expects the activity to have positive outcomes, due to a judgment of limited potential for reinforcement otherwise (Lent et al., 1994). In light of this, the present study may not have been able to increase interest as much as possible, since no significant increases in positive outcome expectations was found, and self-efficacy therefore may not have been able to influence interest fully.

That the simulation was not able the influence the outcome expectations of the students, does not necessarily mean that simulations are not able to influence students’ outcome expectations at all. Instead, it is possible that a single exposure to the simulated lab simply is not enough to alter the students’ preconceptions of what it means to work with real world biology. It may be that if students had multiple occasions to explore the different jobs and tasks within biology, they would be able to slowly adjust their expectations. However, it is also possible that their expectations were quite realistic and accurate beforehand, and that the simulation did not add to their already existing expectations. All students included in the study had chosen to take biology/biotechnology as an elective medium or advanced level subject, indicating that they may already have had positive outcome expectations. Another interesting finding related to outcome expectations, was that the self-evaluative subdomain of outcome expectations was the only subdomain of the three that had a significant unique contribution to interest. This indicates that to have the highest effect on interest development, through outcome expectations, the simulations should focus on giving students learning experiences that enhance their expectations that a career within biology will enable them to achieve their need for personal self-fulfillment. Finally, outcome expectations predicted more of the variance in interest than self-efficacy did, a surprising finding since the social cognitive career theory suggests self-efficacy as the stronger predictor (Lent et al., 1994). This finding is interesting because it may reveal something about the nature of career development in STEM fields. According to Lent et al. (1994) outcome expectations may be more influential in certain contexts, were the outcome of a person’s actions seem unrelated to their own performance, and their self-efficacy appraisals therefore becomes redundant. In this type of context, the person would rely more on their outcome expectations when forming their interests and goals. STEM fields often require a high level of skill from the professionals working with them (European Commission, 2004), so it seems unlikely that students would appraise their own performance as an unimportant factor in the possible outcomes of their actions related to these fields. However, it may be that student’s do not link their judgments of their own performance (i.e., self-efficacy) in biology in high school, with the possible outcomes of actions related to a career within biology, thus making their outcome expectations the more influential mechanism, when forming interest.

In extension to the results found regarding development of interest, the results can be used to make some suggestions about the simulations possible influence on subsequent goal choices and goal actions. The present study did not expect to change student’s goal choices with a single exposure to the learning simulations, and this variable was thus not included in the study. However, because the mechanisms of interest development, proposed by the social cognitive theory was confirmed by the results collected on these variables, it is possible to make cautious considerations of, and suggestions about, the possible effects of the simulation on goal choices, in concordance with the social cognitive career theory. If a teaching method is able to create a learning experience that increases interest through its influence on self-efficacy and outcome expectations, it should also in turn affect goal choices and eventually goal actions. The present results did show the simulations ability to increase both self-efficacy and interest, indicating that they may potentially be able to influence goal choices and eventual goal actions as well in the long term. This is of course only a hypothesis, and the present results do not provide empirical evidence for this. Future longitudinal studies with continued and long term use of the simulations should be conducted to test this hypothesis and provide more solid evidence for the assumption that simulations can influence ultimate career decisions. However, the results do indicate a potential of the simulations to influence academic and career development, and pose a first effort in uncovering the potential use of learning simulations in the future effort of society toward strengthening young students interest in and pursuit of STEM studies and careers.

### Methodological Considerations and Future Research

Some methodological considerations have already been outlined in the previous discussion of the results. This section will provide an overview of these, as well as potential future research directions. The first important methodological limitation of the present study was that it did not test the simulations longitudinally, with multiple uses across time. Most of the findings with regard to the non-cognitive variables, while significant, did not reveal very large effect sizes, and some variables did not change significantly. This may be because a single use of the simulation, or a single lesson for that matter, is not very powerful in changing these types of variables. This is in line with a meta-analysis of simulations for STEM learning, that found higher effect sizes in studies with four to six sessions (D’Angelo et al., 2013). Thus, it is likely that it takes multiple uses to impact self-efficacy, intrinsic motivation, interest and outcome expectations, where each use builds on the previous, serving as a continual and gradual strengthening of the variables. Future research should examine the effect of learning simulations when used across time on multiple occasions, to investigate if the benefits are larger when students have several exposures and time to reflects on and integrate what they learn, with what they already know.

Another important consideration is that the control lessons were not identical and did not necessarily target the knowledge questions equally well. As is evident in the descriptions of the lessons conducted in each high school (see Appendix B for a description of the approach taken by each high school included in the study), the lessons differed somewhat on how many types of exercises and teaching approaches each group was subjected to within the set time frame of 1.5 h. However, results showed no significant differences between the different approaches for gains in knowledge of evolution, self-efficacy, intrinsic motivation, interest, or outcome expectations. The lessons were all based on the content of the simulation and all teachers had the knowledge questions at hand when preparing their lessons, so they should be at least somewhat similar in their content and aim. Furthermore, the aim of the study was to compare the simulation to a traditional lesson (i.e., a lesson similar to what students usually receive), and as such the use of lessons prepared and conducted by the students’ own teachers, using their preferred techniques, provides the best way of doing this. In light of this, results should not be rejected on the basis of this methodological feature, but the relative strength of effects of the interventions compared to each other, should be interpreted with this in mind. In future research it may be beneficial to take this into consideration and streamline the preparation of the control lessons, to make sure they compare more closely to the content of the simulation, and also that they are more similar across schools included in the trial.

In addition to the two methodological considerations, four specific suggestions for future research will be discussed. First, future research should use larger sample sizes to increase the possibility of detecting a potential increases in non-cognitive skills, as the effect of simulations on these varialbes seem to be small to medium in size. Small yet potentially important increases in non-cognitive variables may not be detectable, unless a very large sample size is used. The present study may have missed otherwise significant changes due to limitations in statistical power with a sample size of 128.

Second, future studies should perform a follow-up assessment, to measure the retention of knowledge, as well as the long term effects on non-cognitive variables. This was not possible in the current study due to practical limitations and teacher time constraints. Previous research has found that the knowledge gained from a learning simulation was maintained 40 days after the post-test in a university sample (Bonde et al., 2014), however this should be replicated in other samples, and more research is needed that investigates the retention of the gain in non-cognitive skills like self-efficacy. The effects of the simulation on non-cognitive variables may only be immediate and dilute quickly in the absence of further use of the simulation. Alternatively, the full effect of the simulation may not manifest immediately after the simulation is completed, requiring a certain amount of time for the new experience to assimilate with the students’ previous experiences, judgments, and expectations, meaning that the full effect will not be present immediately after the simulation. Future research with follow-up tests could provide a more wide perspective to the results, investigating the long-term effects of the simulations.

Third, future research should also examine the specific characteristics that make simulations effective by testing different principles of multimedia learning (Mayer, 2009), both with regards to their immediate outcomes, as well as their application toward academic and career development. Furthermore, it would be beneficial to investigate individual and contextual differences, to identify if they influence the simulations efficiency for the individual student, and if there are certain characteristics of the simulations that cater differently to different types of students.

A fourth future research consideration is to replicate this study to assess the generalizability of the results. The simulations used in this study, and thereby the area of application investigated, dealt with a very specific area of science; evolution and biology. Additional research is needed to explore if simulations have similar efficacy in other areas of STEM education, where the teaching methods used may vary considerably (e.g., mathematics).

## Conclusion

Virtual learning simulations are at least as efficient in enhancing learning and self-efficacy as traditional lessons. The findings support the recommendations of de Jong et al. (2013), who emphasized the strength of combining the methods, and the simulations should thus be used in combination with traditional teaching methods, not as a replacement.

In addition, the findings confirmed the assumptions of the social cognitive career theory and revealed that virtual learning simulations may be a useful tool in the effort toward enhancing student’s interest in and goals toward an STEM related career. By utilizing the unique features of the simulations, high schools can provide an accessible and cheap way of giving students a realistic preview of these career paths, and strengthen their self-efficacy, thereby increasing the chance that they ultimately pursue an STEM career.

## Author Contributions

All authors listed, have made substantial, direct and intellectual contribution to the work, and approved it for publication.

## Conflict of Interest Statement

The authors declare that the research was conducted in the absence of any commercial or financial relationships that could be construed as a potential conflict of interest.

## References

[B1] Andrich D. Sheridan B. Luo G. (2010). *Rasch Models for Measurement: RUMM2030.* Perth, WA: RUMM Laboratory.

[B2] Bandura A. (1986). *Social Foundations of Thought and Action: A Social Cognitive Theory*, Vol. 1 Upper Saddle River, NJ: Prentice Hall

[B3] Bandura A. (1999). Social cognitive theory: an agentic perspective. *Asian J. Soc. Psychol.* 2 21–41. 10.1111/1467-839X.00024 11148297

[B4] Bonde M. T. Makransky G. Wandall J. Larsen M. V. Morsing M. Jarmer H. (2014). Improving biotech education through gamified laboratory simulations. *Nat. Biotechnol.* 32 694–697. 10.1038/nbt.2955 25004234

[B5] Bric J. D. Lumbard D. C. Frelich M. J. Gould J. C. (2015). Current state of virtual reality simulation in robotic surgery training: a review. *Surg. Endosc.* 30 2169–2178. 10.1007/s00464-015-4517-y 26304107

[B6] D’Angelo C. Rutstein D. Harris C. Bernard R. Borokhovski E. Haertel G. (2013). *Simulations for STEM Learning: Systematic Review and Meta-Analysis Executive Summary.* Menlo Park, CA: SRI International.

[B7] de Jong T. (2006). Technological advances in inquiry learning. *Science* 312 532–533. 10.1126/science.1127750 16645080

[B8] de Jong T. Linn M. C. Zacharia Z. C. (2013). Physical and virtual laboratories in science and engineering education. *Science (New York, N.Y.)* 340 305–308. 10.1126/science.1230579 23599479

[B9] Deci E. Eghrari H. Patrick B. Leone D. (1994). Facilitating internalization: the self-determination theory perspective. *J. Pers.* 62 119–142. 10.1111/j.1467-6494.1994.tb00797.x 8169757

[B10] Diegelman N. M. Subich L. M. (2001). Academic and vocational interests as a function of outcome expectancies in social cognitive career theory. *J. Vocat. Behav.* 59 394–405. 10.1006/jvbe.2001.1802

[B11] Embretson S. Reise S. P. (2000). *Item Response Theory for Psychologists. Books.Google.com.* Available at: http://books.google.com/books?hl=en&lr=&ie=UTF-8&id=rYU7rsi53gQC&oi=fnd&pg=PP11&dq=ORDERED+LATENT+CLASS+MODELS+IN+NONPARAMETRIC+ITEM+RESPONSE+THEORY&ots=ZAESC95fcK&sig=Rvmsiq0-E7GYnGqw9ejqivzaKd4

[B12] European Commission (2004). *Europe Needs more Scientists.* Brussels: European Commission.

[B13] Fouad N. A. Smith P. L. (1996). A test of a social cognitive model for middle school students: math and science. *J. Couns. Psychol.* 43 338–346. 10.1037/0022-0167.43.3.338

[B14] Furtak E. M. Seidel T. Iverson H. Briggs D. C. (2012). Experimental and quasi-experimental studies of inquiry-based science teaching: a meta-analysis. *Rev. Educ. Res.* 82 300–329. 10.3102/0034654312457206

[B15] Garcia E. (2014). The need to address noncognitive skills in the education policy. *Econ. Policy Inst. Briefing Pap.* 386 1–36.

[B16] Heckman J. J. Kautz T. (2012). Hard evidence on soft skills. *Labour Econ.* 19 451–464. 10.1016/j.labeco.2012.05.014 23559694 PMC3612993

[B17] Heckman J. J. Stixrud J. Urzua S. (2006). The effects of cognitive and noncognitive abilities on labor market outcomes and social behavior. *J. Labor Econ.* 24 411–482. 10.1086/504455

[B18] IBM (2016). *IBM SPSS Advanced Statistics.* Armonk, NY: IBM, 20.

[B19] International Test Comission (2010). *International Test Commission Guidelines for Translating and Adapting Tests. Gefunden Am.* Available at: http://www.intestcom.org

[B20] Labster (2016a). *What is Labster?.* Available at: https://www.labster.com/what-is-labster/

[B21] Labster (2016b). *Labster–Evolution Virtual Lab Simulation.* Available at: https://www.youtube.com/watch?v=vR0fwXOuKss

[B22] Honey M. A. Hilton M. L. (eds). (2011). “Learning science through computer games and simulations,” in *Board on Science Education and Division of Behavioral and Social Sciences and Education* (Washington, DC: The National Academies Press).

[B23] Lent R. W. Brown S. D. Hackett G. (1994). Toward a unifying social cognitive theory of career and academic interest, choice, and performance. *J. Vocat. Behav.* 45 79–122. 10.1006/jvbe.1994.1027

[B24] Lent R. W. Lopez A. M. Lopez F. G. Sheu H.-B. (2008). Social cognitive career theory and the prediction of interests and choice goals in the computing disciplines. *J. Vocat. Behav.* 73 52–62. 10.1016/j.jvb.2008.01.002

[B25] Lent R. W. Lopez F. G. Sheu H.-B. Lopez A. M. (2011). Social cognitive predictors of the interests and choices of computing majors: applicability to underrepresented students. *J. Vocat. Behav.* 78 184–192. 10.1016/j.jvb.2010.10.006

[B26] Makransky G. Bilenberg N. (2014). Psychometric properties of the parent and teacher ADHD rating scale (ADHD-RS): measurement invariance across gender, age, and informant. *Assessment* 21 694–705. 10.1177/1073191114535242 24852496

[B27] Makransky G. Bonde M. T. Wulff J. S. G. Wandall J. Hood M. Creed P. A. (2016a). Simulation based virtual learning environment in medical genetics counseling: an example of bridging the gap between theory and practice in medical education. *BMC Med. Educ.* 16 98 10.1186/s12909-016-0620-6 PMC4807545 27012245

[B28] Makransky G. Thisgaard M. Gadegaard H. (2016b). Virtual learning simulations as preparation for lab exercises: assessing learning of key laboratory skills in microbiology and improvement of essential non-cognitive skills. *PLoS ONE* 11:e0155895 10.1371/journal.pone.0155895 PMC4890735 27253395

[B29] Makransky G. Lilleholt L. Aaby A. (2017). Development and validation of the multimodal presence scale for virtual reality environments: a confirmatory factor analysis and item response theory approach. *Comput. Hum. Behav.* 72 276–285. 10.1016/j.chb.2017.02.066

[B30] Makransky G. Rogers M. E. Creed P. A. (2014). Analysis of the construct validity and measurement invariance of the career decision self-efficacy scale: a rasch model approach. *J. Career Assess.* 2014 1–16. 10.1177/1069072714553555

[B31] Masters G. N. (1982). A rasch model for partial credit scoring. *Psychometrika* 47 149–174. 10.1007/BF02296272

[B32] Mayer R. E. (2009). *Multimedia Learning*, 2nd Edn. New York, NY: Cambridge University Press.

[B33] Pallant J. F. Tennant A. (2007). An introduction to the Rasch measurement model: an example using the Hospital Anxiety and Depression Scale (HADS). *Br. J. Clin. Psychol.* 46 1–18. 10.1348/014466506X96931 17472198

[B34] Peters M. A. Araya D. (2011). Transforming american education: learning powered by technology. *E Learn. Digit. Media* 8 102–105. 10.2304/elea.2011.8.2.102

[B35] Pintrich P. R. R. Smith D. Garcia T. McKeachie W. (1991). A manual for the use of the Motivated Strategies for Learning Questionnaire (MSLQ). *Ann. Arbor. Michigan* 48109 1259.

[B36] Ryan R. M. Deci E. L. (2000). Self-determination theory and the facilitation of intrinsic motivation, social development, and well-being. *Am. Psychol.* 55 68–78. 10.1037/0003-066X.55.1.68 11392867

[B37] Smetana L. K. Bell R. L. (2012). Computer simulations to support science instruction and learning: a critical review of the literature. *Int. J. Sci. Educ.* 34 1337–1370. 10.1080/09500693.2011.605182

[B38] SurveyMonkey (2016). *SurveyMonkey: Free Online Survey Software* & *Questionnaire Tool* San Mateo, CA: SurveyMonkey.

[B39] The Business-Higher Education Forum (2011). *Creating the Workforce of the Future: The STEM Interest and Proficiency Challenge.* Washington, DC: The Business-Higher Education Forum.

[B41] Wang C. Y. Wu H. K. Lee S. W. Y. Hwang F. K. Chang H. Y. Wu Y. T. (2014). A review of research on technology-assisted school science laboratories. *Educ. Technol. Soc.* 17 307–320.

[B42] Williams J. R. (2008). The Declaration of Helsinki and public health. *Bull.* *World Health Organ.* 86 650–652. 10.2471/BLT.08.050955 PMC2649471 18797627

