# Supplementary material for: Virtual Learning Simulations in High School: Effects on Cognitive and Non-cognitive Outcomes and Implications on the Development of STEM Academic and Career Choice
Source: Front Psychol. 2017 May 30;8:805. doi: 10.3389/fpsyg.2017.00805 (PMC5447738; doi:10.3389/fpsyg.2017.00805)
Supplement: Supplementary file 1 [file Appendix_B.PDF]

Appendix B - Description of the lessons specific composition by school

All lessons took 1½ hours.

Middelfart Gymnasium (two classes, 18 & 23 students). One class received a lesson based on a teacher presentation, where students were asked questions when relevant and thereby participated with their own prior knowledge. The other class also had a lesson beginning with a teacher presentation, but additionally dealt in groups with a worksheet prepared by the teacher, using relevant pages in a textbook, followed by a class-based discussion of the worksheet questions.

Nykøbing Katedralskole (two classes, 14 & 19 students). Both classes attended identical lessons. The lesson began with a teacher presentation. After this, students individually read pages from a textbook. When the students finished reading, they worked in groups, answering questions from a worksheet given by the teacher. Finally, the questions were discussed on a class basis.

Odense Tekniske Gymnasium (one class, 22 students). Students were given worksheets with questions about evolution. Students read pages in a textbook and worked in groups of three, discussing the questions, with basis in the textbook. The teacher provided help where needed. The teacher had planned a class discussion in the end of the lesson, but did not have time for this, because the group work took longer than expected.

Ribe Katedralskole (two classes, 17 & 15 students). Both classes attended identical lessons. Students read one of 2 articles about evolution, followed by a group discussion on the basis of teacher set focus points. After this, groups held a short presentation about what they discussed. Finally, the students did an experiment using beans of different color to learn more about natural selection, followed by statistical analysis of the results.

Note: The number of students is the number that were included in the final data analysis, and not the total number of students tested.
