# Supplementary material for: Virtual Learning Simulations in High School: Effects on Cognitive and Non-cognitive Outcomes and Implications on the Development of STEM Academic and Career Choice
Source: Front Psychol. 2017 May 30;8:805. doi: 10.3389/fpsyg.2017.00805 (PMC5447738; doi:10.3389/fpsyg.2017.00805)

Appendix A

1. Video presentation of the Evolution Lab by Labster:

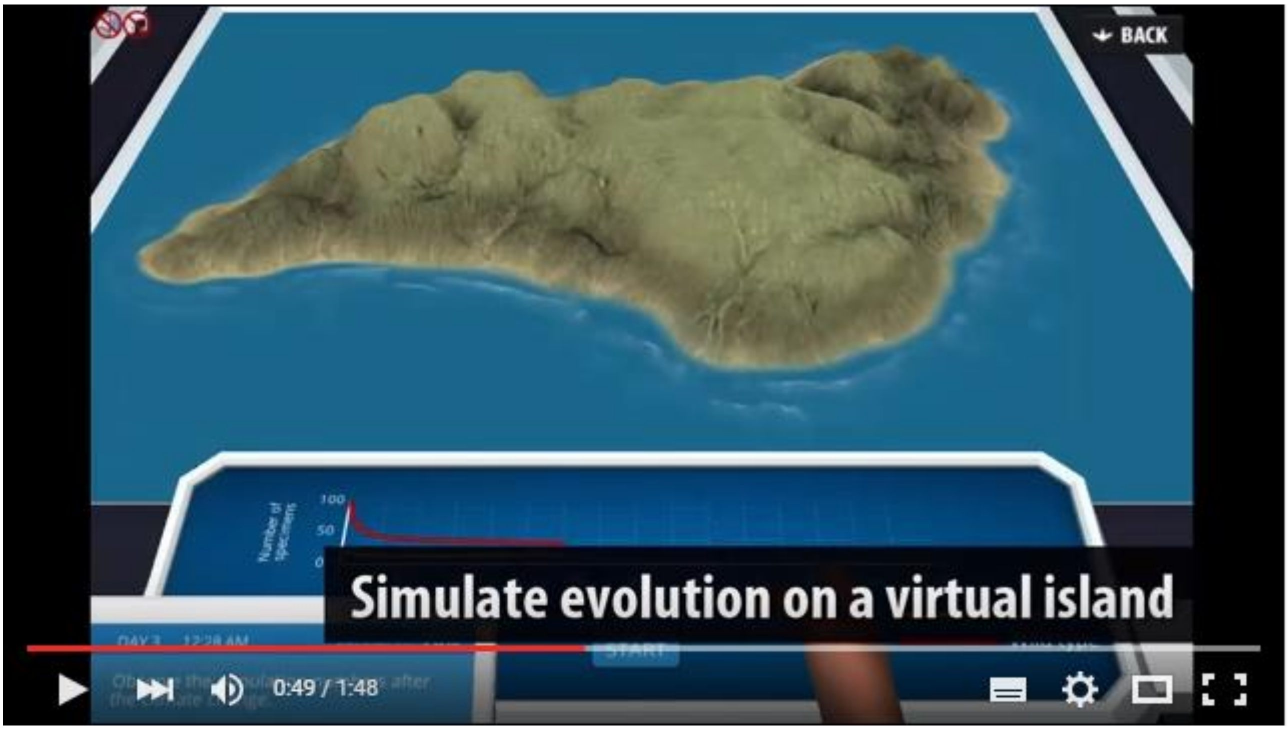

https://www.youtube.com/watch?v=vR0fwXOuKss

2. Screenshot of the evolution lab

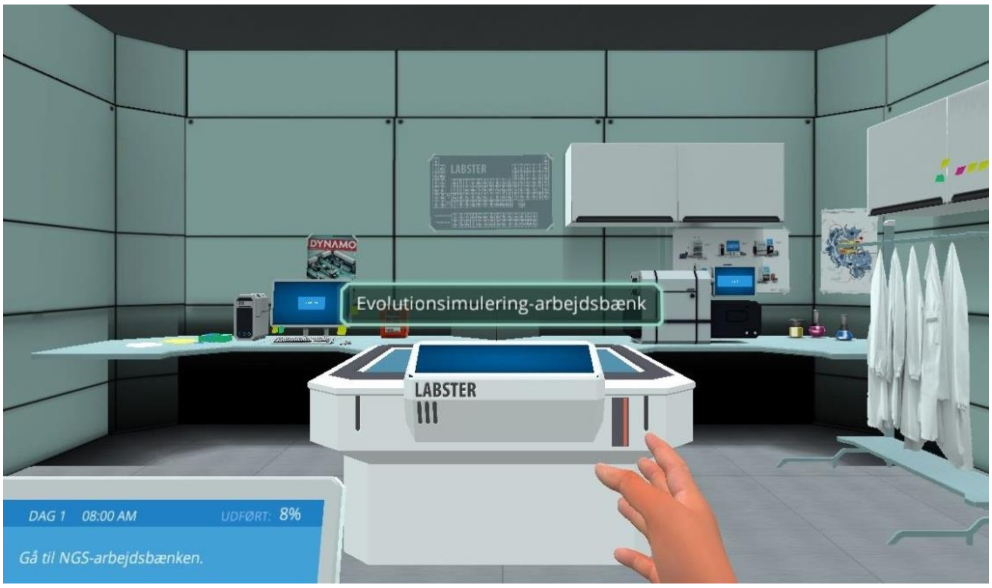

Supplement: Supplementary file 2 [file Appendix_A.PDF]
